# Protocol of trans-Tasman feasibility randomised controlled trial of the Younger Women’s Wellness After Breast Cancer (YWWACP) lifestyle intervention

**DOI:** 10.1186/s40814-022-01114-z

**Published:** 2022-08-02

**Authors:** K. Sharples, N. K. Vear, J. Porter-Steele, D. J. Anderson, T. H. Moeke-Maxwell, B. B. Laing, L. Young, T. G. Bailey, S. Benge, Y. Huang, E. Crowley, R. Day, R. Cartwright, M. Findlay, D. Porter, M. Kuper, I. Campbell, A. L. McCarthy

**Affiliations:** 1 Cancer Trials New Zealand, Auckland, New Zealand; 2 University of Otago, Dunedin, New Zealand; 3 University of Queensland, Brisbane, Australia; 4 Wesley Choices Cancer Support Centre, Brisbane, Australia; 5 University of Technology, Sydney, Australia; 6 University of Auckland, Auckland, New Zealand; 7 Department of Oncology, Auckland Hospital, Auckland, New Zealand; 8 Department of Oncology, Waikato Hospital, Hamilton, New Zealand; 9 Department of Surgery, Waikato Hospital, Hamilton, New Zealand; 10 Mater Research Institute, Brisbane, Australia

**Keywords:** Breast cancer, Feasibility, Acceptability, Translation

## Abstract

**Background:**

Younger women (defined as those < 50 years who are likely pre-menopausal at time of diagnosis) with breast cancer often experience persistent treatment-related side effects that adversely affect their physical and psychological wellbeing. The Women’s Wellness After Cancer Program (WWACP) was adapted and piloted in Australia to address these outcomes in younger women. The aims of this feasibility study are to determine (1) the potential to translate the Younger WWACP (YWWACP) intervention to a broader population base in Aotearoa/New Zealand and Australia, and (2) the potential for success of a larger, international, phase ΙΙΙ, randomised controlled trial.

**Methods:**

This bi-national, randomised, single-blinded controlled trial involves two main study sites in Aotearoa/New Zealand (Kōwhai study) and Australia (EMERALD study). Young women aged 18 to 50 years who completed intensive treatment (surgery, chemotherapy, and/or radiotherapy) for breast cancer in the previous 24 months are eligible. The potential to translate the YWWACP to women in these two populations will be assessed according to several feasibility outcomes. These include examining intervention accessibility, acceptability and uptake; intervention sustainability and adherence; the prevalence components of the intervention in the control group; intervention efficacy; participants’ perception of measurement burden; the effectiveness of planned recruitment strategies; and trial methods and procedures. The studies collectively aim to enrol 60 participants in the intervention group and 60 participants in the control group (total = 120 participants).

**Discussion:**

Ethical approval has been received from the Southern Health and Disability Ethics Committee (Kōwhai ref: 19/STH/215), and Uniting*Care* Human Research Ethics Committee (EMERALD ref: 202103). This study will provide important data on the feasibility of the refined YWWACP in the trans-Tasman context. This study will account for and harmonise cross-country differences to ensure the success of a proposed international grant application for a phase ΙΙΙ randomised controlled trial of this program to improve outcomes in younger women living with breast cancer.

**Trial registration:**

Australian New Zealand Clinical Trials Registry (ANZCTR): Kōwhai ACTRN12620000260921, registered on 27 February 2020. EMERALD ACTRN12621000447853, registered on 19 April 2021.

**Supplementary Information:**

The online version contains supplementary material available at 10.1186/s40814-022-01114-z.

## Background

Younger women (defined as those < 50 years who are likely pre-menopausal at time of diagnosis [1]) treated for breast cancer are a large and growing population, with a worldwide predicted age-standardised incidence rate (ASIR) of 19.7 cases per 100,000 women in 2018 [1]. The ASIR rate was higher in women living in New Zealand and Australia, at 36.7/100,000 [1]. While the survival outcomes of this cohort have improved (> 90% are predicted to still be alive in 5 years) [1], their likelihood of developing treatment-related chronic conditions such as early menopause and abnormal or excessive weight gain is considerable [2]. Psychosocial issues more specific to younger women such as sexual dysfunction, distress, effect on pregnancy and breast feeding, body image disturbance, and disruption of the family and work roles are also significant [3, 4]. Younger women generally have pressing demands on their time, through their social and professional roles, so a flexible program that enables self-management is also essential. The need for support is particularly acute among non-metropolitan women who cannot access face-to-face services due to cost, time, and geographical barriers [5].

In 2017, Australian members of this feasibility study’s research team completed a randomised controlled trial (RCT) of a 12-week whole-of-lifestyle intervention [6]. The intervention was designed to enhance the quality of life of Australian women (*n* = 351) aged > 18 years who had received treatment for a range of cancers, which also enabled them to self-manage their risk of treatment-related chronic conditions (NHMRC/APP1056856). The Women’s Wellness After Cancer Program (WWACP) intervention promoted physical activity, optimal diet, smoking cessation and alcohol reduction, plus strategies for sleep, anxiety and depression, sexual wellbeing, and menopausal symptom management based on the latest research evidence [6]. This multi-modal intervention was delivered via an e-health enabled platform, comprising individual virtually-delivered health professional consultations, an interactive web interface (including podcasts), and an interactive electronic book (iBook). Virtual consultations were delivered by experienced cancer nurses trained in the delivery of the WWACP via Skype or Facetime at baseline, 6 and 12 weeks [6]. It can be delivered flexibly, irrespective of geographic location. Compared to controls, intervention participants reported statistically significant improvements in health-related quality of life [7], i.e., the synergy of factors that contribute to an overall rating of wellbeing [8].

In the course of the RCT, it became apparent that younger women treated for breast cancer had distinct needs that were not entirely met by the intervention (unpublished). This included assessing outcomes related to altered fertility, altered sexual relationships, early menopause, and financial toxicity associated with being unable to work for extended periods during and following treatment. Participants also identified that more simplified instruments were needed, and less variables overall (i.e. less instruments). Therefore, on completion of the trial the intervention was adapted to address these unmet needs and piloted with 40 younger women. The pilot revealed minor problems with the new intervention content, some of the measures used and recruitment approaches, necessitating more refinement of the intervention and methodology and further feasibility testing. Funding for this was subsequently awarded to test the refined Younger WWACP (YWWACP) intervention and study methodology in Aotearoa/New Zealand and Australia, with the collective aim to benefit as many younger women with breast cancer as possible.

The refined intervention commenced feasibility testing via a RCT (Kōwhai study, *n* = 60 women) in Aotearoa/New Zealand in September 2020, with an identical study commencing recruitment in Australia in February 2022 (EMERALD study; *n* = 60 women). Whilst both countries deliver similar cancer care and treatment outcomes, they do have some markedly different cultural nuances, languages, and approaches to research. These two feasibility trials aim to harmonise and consolidate the trial methodology to enable the effective and meaningful pooling, interpretation and evaluation of study outcomes. On completion, we hope to obtain funding to undertake a joint trans-Tasman phase ΙΙΙ RCT to assess the effectiveness of the intervention in improving quality of life and function in younger women with breast cancer.

The aims of the Kōwhai and EMERALD feasibility studies are the following:

1. To determine the potential to translate the YWWACP intervention to a broader population base (i.e. the larger clinical population of which this study is targeting) by examining:
  1.1 The accessibility, acceptability and uptake of the intervention in a representative population of younger women with breast cancer in Aotearoa/New Zealand and Australia, women living with greater deprivation, and women living in non-metropolitan areas.
  1.2 The sustainability of, and adherence to, the intervention over time.
2. To determine the potential for success of a larger, national, randomised controlled trial by examining:
  2.1. Participants’ perceptions of measurement burden.
  2.2. The effectiveness of the planned recruitment strategy.
  2.3. The prevalence components of the intervention in the control group.
  2.4. Intervention efficacy (i.e. reduced waist circumference).
  2.5. Trial methods and procedures (including randomisation and follow-up procedures).

Potential outcomes include determination of a feasible approach for a multi-site trial in different cancer contexts, and confirmation that the YWWACP has been appropriately adapted to promote uptake, adherence and efficacy. We also plan to carry out a meta-analysis of the patient-reported outcomes to inform the phase III trial, including the calculation of the target sample size (not included in this protocol).

## Methods/design

**Fig. 1:**
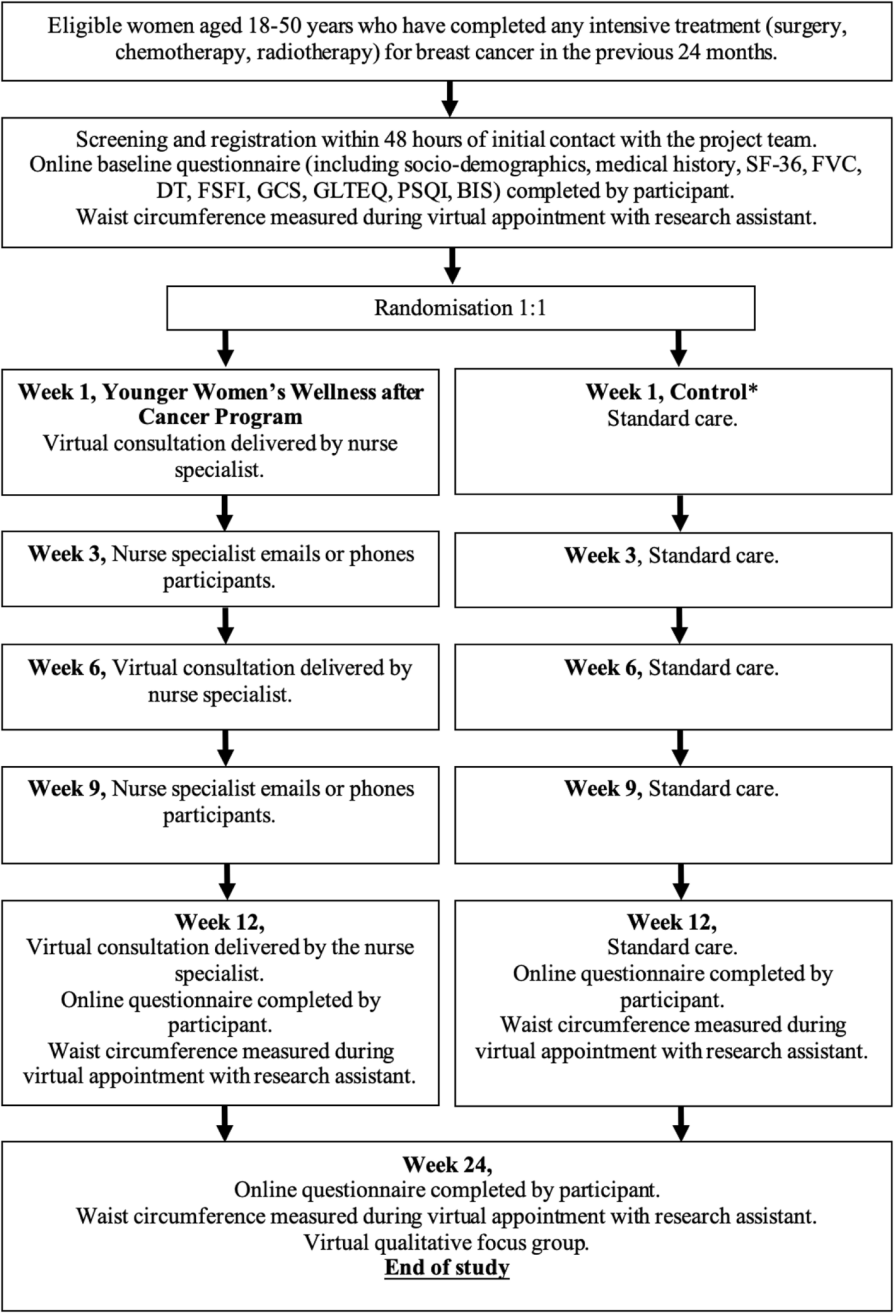
Kōwhai and EMERALD Feasibility Trial Schema. * Receive general information from their usual health professionals during clinic visits about the management of all symptoms. This includes the general advice available to them about physical activity, diet, tobacco and alcohol abstinence, plus information about support services (relevant to their country)

**Fig. 2:**
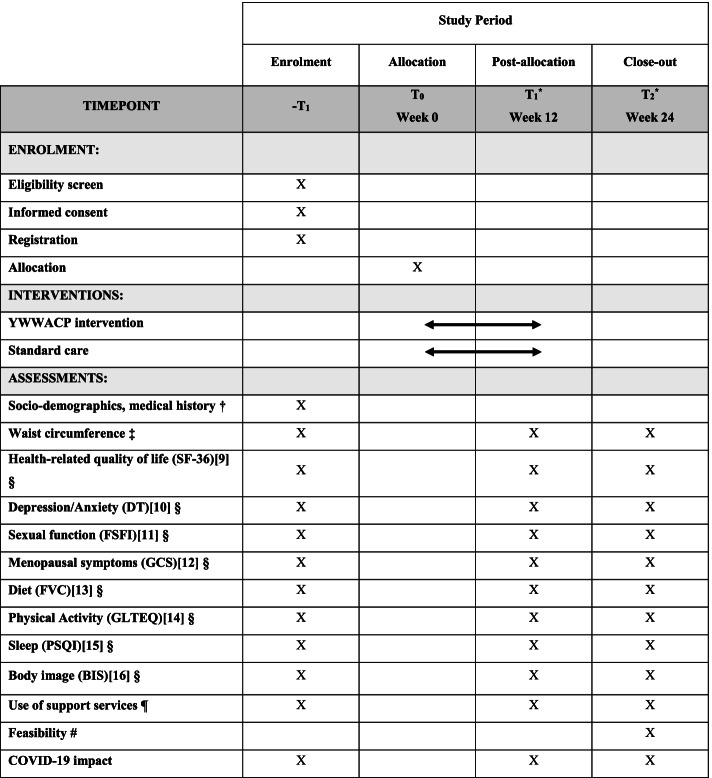
SPIRIT Flow Diagram of Schedule of Enrolment, Intervention and Assessments. ^*^A window of up to 14 days is allowed for randomisation following recruitment, and for collection of outcome data. ^†^Age, ethnicity, relationship and employment status, income, deprivation, tumour stage and type, cancer treatments received, ongoing medication, comorbidities. Entered by patient directly into online platform. ^‡^Waist circumference is measured by a research assistant in a virtual appointment. ^§^Patient-reported outcome (PRO) measures: SF-36—Short Form-36; DT—Distress Thermometer; FSFI—Female Sexual Function Index; GCS—Greene Climacteric Scale; FVC—Food Variety Checklist; GLTEQ—Godin Leisure-Time Exercise Questionnaire; PSQI—Pittsburgh Sleep Quality Index; BIS—Body Image Scale. Entered by the patient directly into online platform. ^¶^Self-reported use of support services and interventions offered. ^#^Free text feasibility survey

The feasibility studies described here follow the planned methods for the phase III trial. They are randomised, single-blinded controlled trials, one in Aotearoa/New Zealand (Kōwhai study) and the other in Australia (EMERALD study). The protocols for the two studies are identical apart from some site-specific details as outlined below. Kōwhai is funded by the Health Research Council of New Zealand (19/622), and EMERALD by the Wesley Medical Research Institute (ID2020CR02). Ethical approval was received from the Southern Health and Disability Ethics Committee (Kōwhai ref: 19/STH/215), and Uniting*Care* Human Research Ethics Committee (EMERALD ref: 202103). Both are prospectively registered with the Australian New Zealand Clinical Trials Registry (Kōwhai: ACTRN12620000260921; EMERALD: ACTRN12621000447853). The study flow is provided in Fig. 1, with a SPIRIT flow diagram provided in Fig. 2.

All amendments to the studies (protocol, participant information sheet) will be approved by the relevant ethics committee prior to being implemented. Approved changes will be communicated to the research team and participants (as per the information in the participant information sheet and consent form).

### Screening and recruitment

Both studies aim to recruit young women aged 18 to 50 years who completed intensive treatment (surgery, chemotherapy, and/or radiotherapy) for breast cancer in the previous 24 months. Participants can be on endocrine or maintenance therapy during the study period. Further inclusion criteria include access to the internet on a computer, smartphone, or tablet device; and able to speak and read English. Exclusion criteria include advanced or metastatic disease, and/or any clinical contraindication that precludes safe completion of the program in the judgement of the clinical members of the project team.

To promote recruitment, we work with our established networks of clinicians at participating health services (Auckland, Waikato, and Brisbane Hospitals) and cancer support groups in Aotearoa/New Zealand (Cancer Society Auckland and Northland Divisions, Breast Cancer Foundation New Zealand, Breast Cancer Coalition Aotearoa, and Pinc & Steel New Zealand) and Australia (Wesley Choices Cancer Support Centre Australia). Women are invited to express interest in the study through a link to an online platform, following which they are provided with detailed information and the opportunity to talk to a member of the study teams. Women who are eligible and provide electronic consent (consent forms provided in Additional files 1 and 2) are registered and their baseline data collected.

### Randomisation

Eligible, consenting participants with complete baseline data are randomly allocated 1:1 to receive standard care (control) or the YWWACP intervention in the electronic data capture system used by Cancer Trials New Zealand (CTNZ). Randomisation should take place within 14 days after registration. The web-based randomisation process is carried out by a member of the research team who is independent of recruitment and data collection. Randomisation is blocked over time, using random block sizes to aid concealment of allocation. This is a single-blinded study, where only the research assistant (RA) is blinded to the assigned treatment. The RA instructs participants on how to conduct waist measurements at baseline (*T*_0_), weeks 12 (*T*_1_), and 24 (*T*_2_) and is blinded to ensure no influence of group allocation on instructions given. No other study investigators are blinded. This means emergency unblinding is not required.

### Intervention

The 12-week intervention targets health education and health promotion incorporating international recommendations for the core outcomes of physical activity, diet, and minimising alcohol and abnormal or excessive weight gain, as well as strategies to manage smoking cessation, sleep, distress, relationship and body image concerns, menopausal symptoms, and sexual and emotional wellbeing over a 12-week period. The timing and application of these strategies reflects the particular life stage of these women and is personalised to meet participants’ individual goals and functional capacities (Table 1).

**Table 1:** Intervention—Younger Women’s Wellness After Cancer Program

Behaviour	Recommendations	Rationale
Physical activity	Be moderately physically active, equivalent to brisk walking, for ≥ 30 min daily. As fitness improves, aim for ≥ 60 min of moderate (or for ≥ 30 min of vigorous) physical activity every day.	Physical activity of longer duration or greater intensity is more beneficial. All forms of physical activity protect against some cancers, as well as against abnormal or excessive weight gain.
Diet	Eat mostly foods of plant origin. Limit consumption of energy dense foods. Avoid sugary drinks. Limit intake of red meat and avoid processed meat.	The evidence indicates that most diets that are protective against cancer mainly comprise foods of plant origin. Energy-dense foods and sugary drinks contribute to abnormal or excessive weight gain. The evidence also indicates foods of animal origin are nourishing and healthy if consumed in modest amounts.
Alcohol	If alcoholic drinks are consumed, limit consumption to no more than one drink per day.	The evidence on balance justifies alcohol abstinence, although some evidence indicates that modest amounts of alcohol could reduce the risk of coronary heart disease.
Abnormal or excessive weight gain	Be as lean as possible within the normal weight range. Avoid weight gain and increases in waist circumference.	Maintenance of a healthy weight could be one of the most important ways to protect against treatment-related chronic diseases.

Source: World Cancer Research Fund and American Institute for Cancer Research (WCRF/AICR). Continuous Update Project Report Summary: Food, Nutrition and Physical Activity and the Prevention of Breast Cancer 2018

The intervention content is delivered via a hardcopy book, an interactive electronic book (program journal), an interactive web interface (including podcasts) and a series of health professional consultations for tailored education and coaching (delivered virtually). See Table 2 for intervention content and delivery strategies.

**Table 2:** Younger Women’s Wellness After Cancer Program Intervention Content and Delivery Strategies

Week/s	Delivery strategies	Content
1	Individual or group virtual consultation delivered by cancer nurse as preferred by participants	Virtual consultation delivered by specialist cancer nurse: Phone coaching, iBook/PDF/hardcopy, health education material, website and email. Introduction to website and program. Development of tailored health education based on agreement between nurse and participant on an individualised plan and goals. Discuss healthy weight measures and associated risk factors, i.e. waist circumference, waist/hip ratio. Discuss exercise, physical activity, healthy eating, and other concerns and appropriate screening.
3, 9	Email/phone	At weeks 3 and 9, a follow-up email is sent from the consultation nurse and zoom consultation if requested to the participant. This phase of communication enquires how the participant is progressing with the program, reviews their individualised plan/goals, identifies barriers and plans for completion of the program.
6	Virtual consultation delivered by nurse	Through health education and motivational interviewing, this virtual appointment addresses: • Review of plan and goals • Behavioural relapse prevention strategies • Answer questions and deliver any additional program materials required (i.e. menopause, stress, sleep). Participant goals set in the first consultation will be reviewed and revised as necessary, including discussion of a personal action plan and identification of barriers. Issues or concerns raised by the participant addressed by nurse.
12	Virtual consultation delivered by nurse	Discussion and review of how the participant found the program and whether individual goals were met including waist circumference measures. The participant encouraged to keep up positive behaviour change following the formal program. Future goals and relapse prevention discussed to encourage sustainability.

The hardcopy book brings together lifestyle information to assist with commencing and/or maintaining a balanced lifestyle following treatment for breast cancer. It includes a week-by-week, stepped program divided into four main components [(1) changing lifestyle; (2) maintaining habits, (3) healthy screening, and (4) moving forward]. The book is used alongside the virtual consultations with the cancer nurse specialist and the program journal. The program journal provides all necessary health promotion content and supports participants to log relevant health and lifestyle information into the journal. A weekly physical activity planner encourages participants to plan ahead for their physical activity in the following week. The interactive website incorporates news and announcements; new evidence; an online moderated ‘community’ allowing participants to communicate with each other; and an overview of the program. The website is adapted for all computing platforms, including smartphones and tablet devices.

Three consultations with a cancer nurse specialist trained in the intervention are conducted via videoconferencing. In keeping with privacy guidelines, no recording of the consultations is undertaken. The nurse specialist measures intervention adherence at the consultations as well as resources accessed at weeks 12 (*T*_1_) and 24 (*T*_2_).

Modification of the intervention is permitted if deemed necessary by the trained nurse specialist at any time during the intervention period. The reasons for modifications are captured. Discontinuation of the intervention can occur if the lead investigator decides the participant is at risk (e.g. serious medical condition, pregnancy), if the participant is unable to complete the program or fails to comply, and/or if the participant withdraws their consent. Reasons for discontinuation of the intervention are recorded.

### Standard care

Participants allocated to the standard care group receive general information from their usual health professionals during routine clinic visits about the management of all symptoms. This includes general advice available regarding physical activity, diet, tobacco and alcohol abstinence, plus information about support services such as Pinc & Steel (physiotherapy and exercise) and the Divisions of the Cancer Societies in Aotearoa/New Zealand, and Cancer Council Queensland in Australia. Participants’ use of these services, and any interventions they offer, are recorded. Control participants are offered a hard copy of the YWWACP book on completion of the study.

### Outcome measures

The feasibility study collects both the efficacy outcome measures for the planned phase III trial (in order to test feasibility) as well as specific feasibility study outcome measures. There are three data collection time points; baseline (*T*_0_), 12 weeks (*T*_1_), and 24 weeks (*T*_2_). A window of up to 14 days is allowed for *T*_1_ and *T*_2_ (Fig. 2).

#### Efficacy outcome measures

The efficacy patient-reported outcome (PRO) measures are collected electronically by the e-PRO platform. They include health-related quality of life (Short Form-36 [9]), distress (Distress Thermometer [10]), sexual function (Female Sexual Function Index [11]), menopausal symptoms (Greene Climacteric Scale [12]), diet (Food Variety Checklist [13]), physical activity (Godin Leisure-Time Exercise Questionnaire [14]), sleep (Pittsburgh Sleep Quality Index [15]), and body image (Body Image Scale [16]). Waist circumference is also measured.

#### Feasibility outcome measures

Objective 1.1—Assess the accessibility, acceptability and uptake of the intervention:

- Content analysis of qualitative data (collected via qualitative interview and semi-structured feasibility questionnaire) to assess the acceptability, accessibility, and uptake of the intervention.
- Socio-demographics (ethnicity, level of deprivation, and rurality) of participants and non-participants (as recorded in study screening log data).
- Reasons for non-participation (as recorded in study screening log data).

Objective 1.2—Assess the sustainability of, and adherence to, the intervention over time:

- Content analysis of qualitative data collected (qualitative interview and semi-structured feasibility questionnaire) to assess the sustainability of, and adherence to, the intervention over time.
- Proportion of participants who discontinue the intervention (as recorded by nurse specialist).
- Reasons for intervention discontinuation (as recorded by nurse specialist).

Objective 2.1—Assess participants’ perceptions of measurement burden:

- Content analysis of qualitative data collected (qualitative interviews and semi-structured feasibility questionnaire) to assess measurement burden.
- Time taken to complete questionnaires (as self-reported by participant in semi-structured feasibility questionnaire).
- Questionnaire completion at weeks 12 (*T*_1_) and 24 (*T*_2_) (by single measure and all measures combined).

Objective 2.2—Assess the effectiveness of the planned recruitment strategy:

- Proportions of consenting participants registered on the study.
- Reasons for non-registration/randomisation (as recorded in study screening log).

Objective 2.3—Assess the prevalence components of the intervention in the control group:

- Self-reported access to support services, and use of any interventions offered.

Objective 2.4 Assess Intervention efficacy

- Waist circumference.

Objective 2.5—Assess trial methods and study processes (including recruitment, randomisation, and follow-up processes):

- Time to informed consent from submission of participant expression of interest.
- Time to randomisation from study registration.
- Time to randomisation from completion of baseline assessments.
- Time to intervention access from randomisation.
- Proportion of patients randomised who complete week 12 (*T*_1_) assessments.
- Proportion of patients randomised who complete week 24 (*T*_2_) assessments.

Using the above objectives, feasibility of the main study evaluating the YWWACP will be determined if the feasibility study:

- Achieves proportional representation in the study population (Objective 1.1),
- Reaches a target participation rate of 80% at 12 weeks, and 50% at 24 weeks [] (Objective 1.2),
- Achieves a questionnaire completion rate of above 80% (Objective 2.1),
- Recruits to target (120 participants) within the target timeframe (18 months) (Objective 2.2),
- Reports the use of resources/programs similar to the YWWACP in the control arm of less than 30% (Objective 2.3),
- Assesses changes in waist circumference (but this will not determine the decision on whether to move forward with the larger trial as it is not a key outcome) (Objective 2.4), and
- Collects information from outcomes listed in objective 2.5 that can be used to refine study methods for the larger, main trial (Objective 2.5).

Data on demographic characteristics and medical history are entered by the patient directly into the-online e-PRO platform. Participants are provided with access to the e-PRO platform to complete the PRO measures, and are sent email messages, as appropriate, to prompt completion of questionnaires at the required time points. In the event that a participant is unable to access the e-PRO online (e.g. technical fault), paper questionnaires will be mailed to the participant for completion. Waist measurements will be taken in triplicate during a virtual consultation with the RA. The RA will adhere to World Health Organisation standardised protocols [17] for the collection of waist circumference measurements for this study. A tape measure will be supplied to the participant to be used at all measurement appointments.

Intervention participants will complete a free text questionnaire at week 24 (*T*_2_) asking them to comment on the following: time taken to complete questionnaires; strengths and/or weaknesses of the intervention; cultural relevance of the intervention; experience of accessing the intervention; perceptions of the digitally delivered nurse coaching sessions; any suggested improvements in study design or intervention. Focus groups will be conducted virtually to collect feasibility data verbally. Information is obtained from both intervention and control groups on use of prescription medications, herbal medicines and cancer support services, and the impact of COVID-19 on participation through an online feasibility questionnaire at weeks 12 (*T*_1_) and 24 (*T*_2_).

#### Safety measures

All adverse events (AE) occurring after the date of consent are reported as AE and graded using criteria established by the National Cancer Institute (NCI) CTCAE version 5. Women who become pregnant during the trial will be advised to discontinue the intervention. Serious AE (SAE) must be reported to the study manager within 24 h of becoming aware of the event. Relapse and death due to breast cancer are not considered to be SAE. The reporting requirement for SAE applies for all events occurring up to 28 days after the last administration of study intervention. All unresolved AE will be followed by the study team until resolved, the participant is lost to follow up, or the AE is otherwise explained.

### Quality assurance

The nurse specialist is fully trained in intervention delivery and adheres to documented policies and procedures. Case review by the study manager of at least one virtual consultation per month monitors adherence to study protocols, with the nurse completing a checklist at the end of each session to indicate the strategies used.

Oversight of the studies is provided by a single Trial Management Committee (TMC), which is responsible for funding and setup, ongoing study conduct, promotion of the study and the interpretation of results at both sites. Both studies have the same statistics and database team, and use the same data systems. The Kōwhai study is managed by the CTNZ trial coordinating centre and the EMERALD study is managed by the Wesley Choices Cancer Support Centre in conjunction with the Wesley Research Institute. Each trial coordinating centre follows standard operating procedures for safety reporting, and to ensure quality and adherence to Good Clinical Practice. Data management and statistical support is provided by CTNZ for both trials.

The RAs receive data collection training and are audited monthly. Participants who withdraw from the study are encouraged to provide follow-up data. Information on reasons for withdrawal are collected. Details for participant compensation as part of this trial are provided in the participant information sheets (provided in Additional files 1 and 2).

Data are entered into the e-PRO platform by the participant or by a member of the study team, as appropriate. Only specific research team members are authorised to access the electronic platform, using a unique user ID and a password. The system also promotes retention and follow-up as it emails reminders to the participants and alerts the research staff to study tasks (e.g. organisation of 12-week appointments).

### Data analysis

Analysis will follow standard approaches for descriptive analyses. For each trial, proportions, means and standard deviations (little skew), or medians and interquartile ranges (serious skew), will be reported as appropriate, and 95% confidence intervals will be provided for proportions and means/medians. Waist circumference will be compared in intervention and control arms using a linear mixed model with a treatment-time interaction. All analyses will be carried out in Statistical Analysis System (SAS) or STATA.

#### Sample size calculation

For each of the trials (Kōwhai and EMERALD) a sample of 60 patients was chosen so that proportions between 0.1 and 0.9 could be estimated with a margin of error < 0.13.

## Discussion

Women with breast cancer have been involved in the design (content and delivery method) of the intervention and the research method from conceptualisation. This included semi-structured focus groups with 16 women less than 2 years following acute treatment for breast cancer (< 40 years, *n* = 6; > 40 years, *n* = 10) [18] and a cross-sectional survey study in 85 women who had finished menopause-inducing cancer treatment at least three months prior (average age = 45 years; breast cancer = 54% of total sample) [19]. Twenty-two participants from the cross-sectional survey study (average age = 44 years; breast cancer = 41% of total sample) further participated in qualitative interviews [5]. This series of studies determined the content and format of the original WWACP. CI Young is also a consumer representative highly trained in the conduct of clinical trials and has consistently advised from trial inception on all aspects of the study’s methodology, including the formulation of research endpoints, recruitment strategies and choice of outcome measures. The study methodology was tested in the Australian pilot, with feedback from participants indicating a significant survey burden (average 90 min to complete at each time point). As a result of this feedback, the survey instruments were changed to ensure that, while each construct is reliably and validly measured in the study described in this paper, the survey suite is much shorter (designed for an average 30 min to complete at each time point). Free text responses at week 12 (*T*_1_) will ask participants to provide their perceptions of the content of the new measurement suite and the time taken to complete it. In addition, the content of the intervention was initially developed after a series of focus groups with women with breast cancer and has been refined with their feedback from pilot work in both countries. In terms of conducting the study, consumer representatives in both study sites actively participate in the monthly TMCs. The study results will be disseminated to all participants at their request in a lay report, the content and format of which will be designed with the consumer representatives of the TMC.

A limitation of this study is participants needing to speak English, which could exclude some First Nations women or individuals from other language groups from participating in the Kōwhai or EMERALD studies. It should be noted, however, that we are currently working with Māori women (First Nations Aotearoa/New Zealand) and Cantonese women in Hong Kong to develop culturally adapted interventions and research protocols to ensure their culturally specific needs are addressed in further iterations.

There have been inevitable delays associated with COVID-19 lockdowns after receiving funding. We are fortunate that to date these have not had a significant impact in Aotearoa/New Zealand, where the study has been vigorously championed by our partners and enthusiastically received by the target group, even when an extended lockdown was ordered contemporaneous with study commencement. The Aotearoa/New Zealand study commenced in September 2020, 7 months after the intended start date in late February. Target baseline recruitment of 60 women was completed in March 2021 (consistent with our original timeline despite COVID-19); 12-week intervention delivery was completed in July 2021 and longitudinal assessments were completed in September 2021. We expect the same rapid uptake in the Australian site once it commences. Our preliminary work when developing this program indicated an enormous appetite for this type of intervention and we have the advantage in a research context dominated by COVID-19 that the intervention, consultation, and data collection procedures are entirely virtually delivered. In addition, the intervention is easily adapted to lockdown conditions, as it encourages creative and sustainable self-management of participants’ lifestyle concerns. The Australian feasibility study commenced in March 2022.

## Supplementary Information

**Additional file 1.** Kōwhai Study Participant Information Sheet and Consent Form

**Additional file 2.** EMERALD Study Participant Information Sheet and Consent Form

## Data Availability

As per funding body requirements, deidentified data may be made publicly available to other researchers subject to requirements of indigenous data sovereignty.

## Abbreviations

AE: Adverse events
ANZCTR: Australian New Zealand Clinical Trials Registry
ASIR: Age-standardised incidence rate
CI: Chief investigator
CTNZ: Cancer Trials New Zealand
e-PRO: Electronic patient-reported outcome
NCI: National Cancer Institute
NHMRC: National Health and Medical Research Council
PRO: Patient-reported outcome
RA: Research assistant
RCT: Randomised controlled trial
SAE: Serious adverse events
SAS: Statistical Analysis System
TMC: Trial Management Committee
WWACP: Women’s Wellness After Cancer Program
YWWACP: Younger Women’s Wellness After Cancer Program
