# Supplementary material for: Protocol of trans-Tasman feasibility randomised controlled trial of the Younger Women’s Wellness After Breast Cancer (YWWACP) lifestyle intervention
Source: Pilot Feasibility Stud. 2022 Aug 2;8:165. doi: 10.1186/s40814-022-01114-z (PMC9343821; doi:10.1186/s40814-022-01114-z)
Supplement: Supplementary file 2 — Additional file 2. EMERALD Study Participant Information Sheet and Consent Form [file 40814_2022_1114_MOESM2_ESM.docx]

# Additional file 2. EMERALD Study Participant Information Sheet and Consent Form
